# Genome-wide characterization and analysis of WRKY transcription factors in *Panax ginseng*

**DOI:** 10.1186/s12864-021-08145-5

**Published:** 2021-11-18

**Authors:** Peng Di, Ping Wang, Min Yan, Peng Han, Xinyi Huang, Le Yin, Yan Yan, Yonghua Xu, Yingping Wang

**Affiliations:** 1 State Local Joint Engineering Research Center of Ginseng Breeding and Application, College of Chinese Medicinal Materials, Jilin Agricultural University, 130118 Changchun, China; 2 State Key Laboratory Breeding Base of Dao-di Herbs, National Resource Center for Chinese Materia Medica, China Academy of Chinese Medical Sciences, 100700 Beijing, China

**Keywords:** Transcription factors, WRKY domain, *Panax ginseng*, Ginsenosides

## Abstract

**Background:**

*Panax ginseng* is a well-known medicinal plant worldwide. As an herbal medicine, ginseng is also known for its long lifecycle, which can reach several decades. WRKY proteins play regulatory roles in many aspects of biological processes in plants, such as responses to biotic or abiotic stress, plant development, and adaptation to environmental challenges. Genome-wide analyses of WRKY genes in *P. ginseng* have not been reported.

**Results:**

In this study, 137 PgWRKY genes were identified from the ginseng genome. Phylogenetic analysis showed that the PgWRKYs could be clustered into three primary groups and five subgroups. Most of the PgWRKY gene promoters contained several kinds of hormone- and stress-related cis-regulatory elements. The expression patterns of PgWRKY genes in 14 different tissues were analyzed based on the available public RNA-seq data. The responses of the PgWRKY genes to heat, cold, salt and drought treatment were also investigated. Most of the PgWRKY genes were expressed differently after heat treatment, and expression trends changed significantly under drought and cold treatment but only slightly under salt treatment. The coexpression analysis of PgWRKY genes with the ginsenoside biosynthesis pathway genes identified 11 PgWRKYs that may have a potential regulatory role in the biosynthesis process of ginsenoside.

**Conclusions:**

This work provides insights into the evolution, modulation and distribution of the WRKY gene family in ginseng and extends our knowledge of the molecular basis along with modulatory mechanisms of WRKY transcription factors in ginsenoside biosynthesis.

**Supplementary Information:**

The online version contains supplementary material available at 10.1186/s12864-021-08145-5.

## Background

Transcription factors (TFs) bind to the DNA regulator sequence to modulate the rate of gene transcription, and they usually play an essential role in responding to complex environmental changes during plant development [1, 2]. The WRKY family is a prominent TF family in higher plants [3] that regulates various biological functions [4]. The name of WRKY TFs derives from their DNA binding domain, which usually contains a polypeptide sequence consisting of WRKYGQK, as well as a zinc finger motif, either C2H2 (C-X4–5-X22–23-H-X1-H) or C2HC (C-X7-C-X23-H-X1-C) [5, 6]. In plants, the WRKY family has three primary groups (Groups I, II, and III) based on the WRKY domain numbers along with the zinc-finger motif [5, 7]. Group I WRKYs harbor two WRKY domains; group II and III WRKYs carry only one WRKY domain. Group II WRKYs are usually categorized into five subgroups, named IIa, -b, -c, -d, and -e, on the basis of their phylogenetic relationships [5]. The differences between Group III and II WRKY proteins are the type of zinc finger motifs; the zinc finger is C-X4-5-C-X22-23-H-X1-H in group II and C-X7-C-X23-H- X1-C in group III [8].

There are 75 WRKY genes in *Arabidopsis thaliana* [9], 182 WRKY genes in soybean (*Glycine max*), 102 WRKY genes in cotton (*Gossypium hirsutum*), and 98 WRKY genes in rice (*Oryza sativa*) [10–12]. The first WRKY gene was discovered from sweet potato (*Ipomoea batatas*) in 1994 [13]. Since then, much research has been carried out on the functions of WRKY. To date, the study of the WRKY family has mainly addressed the biological progress of plants, consisting of biotic and abiotic stress responses, along with plant growth processes. Recent studies have shown that the WRKY family participates in many developmental processes of the plant, such as root hair development [14], pollen development [15], growth types [16], flowering time [17], fruit ripening [18], and leaf senescence [19–21]. Especially in response to abiotic and biotic stresses, WRKY usually plays an indispensable role. It modulates responses to drought stress in *A. thaliana* [22, 23] and heat stress in rice (*O. sativa*) [24], salt stress in soybean (*G. max*) [25], ozone stress in *Viburnum lantana* and *Pak Choi* [26, 27], and cold stress in rice (*O. sativa*) [28, 29].

*Panax ginseng*, as the most famous Panax species around the world, has an over 5000-year history of medicinal use in East Asia [30]. The market for ginseng reaches over 2 billion USD each year [31]. Ginseng is a shade-requiring perennial herb that belongs to the Araliaceae family. The growth and development of wild ginseng are relatively slow, and its maximum age can reach 100 years [32]. During its lifespan, wild ginseng is highly exposed to harsh environmental conditions and has to respond to diverse abiotic or biotic stresses. As the key TFs in the plant stress response system, WRKY family genes should play an important role in ginseng growth and development. A previous study investigated WRKY genes in ginseng adventitious roots based on transcriptome analysis [33, 34]. However, the transcriptome results only present parts of the WRKY family genes, and most of the WRKY gene family is still unidentified from the ginseng genome. The expression trend of the WRKY family in different parts and at different developmental ages and the response trend to abiotic stress are still unknown.

Herein, 137 ginseng WRKY genes were identified, and these PgWRKYs can be further categorized into three major groups with five subgroups. The evolutionary relationship of PgWRKY genes was explored. After that, the gene structure of PgWRKYs was elucidated. The expression profile of PgWRKY genes in diverse tissues and response trends for many abiotic treatments were also studied. Based on the tissue-specific expression and coexpression analysis of ginsenoside pathway genes, 11 PgWRKYs were selected as potentially modulatory TFs involved in the ginsenoside biosynthetic pathway. All of these data reveal essential information for subsequent functional study of WRKY gene family members in *P. ginseng*.

## Results

### Identification, classification and phylogenetic analysis of WRKY transcription factors in ginseng

A total of 137 WRKY (PgWRKY1 to PgWRKY137) transcription factors were identified from the public ginseng genome data resource and then confirmed by the HMMSCAN search, SMART database, and NCBI-Conserved Domain Database. The full data for these genes consisting of gene name, gene ID, protein length, gene locus numbers, gene length, coding sequences (CDS) length, molecular weight (MW) and isoelectric point (PI) are shown in Table S1. The lengths of these WRKY genes varied from 465 to 21,240 bp. The protein length ranged from 124 to 1303 amino acids (AAs), the MW ranged from 13.9 to 143.53 kDa, and the pI varied from 4.12 to 10.76.

**Fig. 1:**
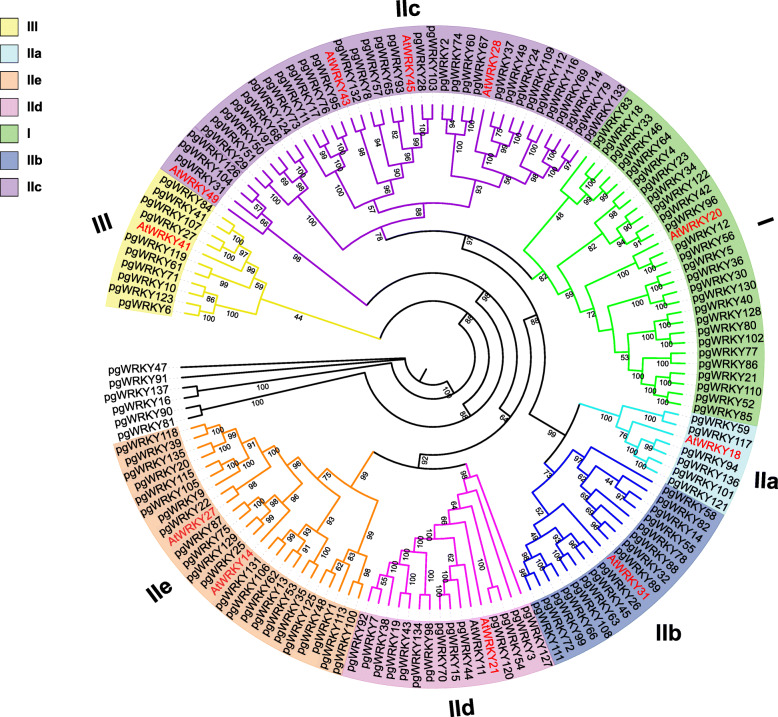
Phylogenetic analysis of ginseng WRKY proteins. The ML tree was constructed in line with WRKY protein sequences alignment. The percentage of replicate trees are presented along branches, and they were computed via bootstrap tests 1000 replicates for reliability verification

An unrooted phylogenetic tree with 137 ginseng WRKYs using maximum likelihood (ML) methods (Fig. 1) was constructed to classify and explore the evolutionary relationship of PgWRKY genes. The AtWRKYs classification was used as the reference. All 137 PgWRKY genes were categorized into three primary groups and five subgroups in group II. The 27 PgWRKYs harboring two WRKY domains were clustered into Group I. Group II constituted 95 WRKYs having a single WRKY domain along with a zinc finger motif of C-X4–5-C-X23-H-X1-H. Furthermore, group II was stratified into five subgroups based on the existence of distinct sequences on their zinc finger motifs. Subgroup IIa harbored a CX5CPVKKK(L/V)Q motif, subgroup IIb contained a CX5CPVRKQVQ motif, subgroup IIc harbored a CX4C motif, subgroup IId included a CX5CPARKHVE motif, and subgroup IIe contained a CX5CPARK(Q/M)V(E/D) motif. Six WRKY proteins were classified as IIa, 16 as IIb, 35 as IIc, 15 as IId, and 23 as IIe. Ten PgWRKYs harboring a single WRKY domain along with a C2HC zinc finger motif (C-X7-C-X23–31-H-X1-C) were assigned to group III. Six PgWRKYs (PgWRKY47, -91, -137, -16, -90, -81) were not categorized into any group even though they harbored a WRKY domain along with a C2H2-type zinc-finger motif.

**Fig. 2:**
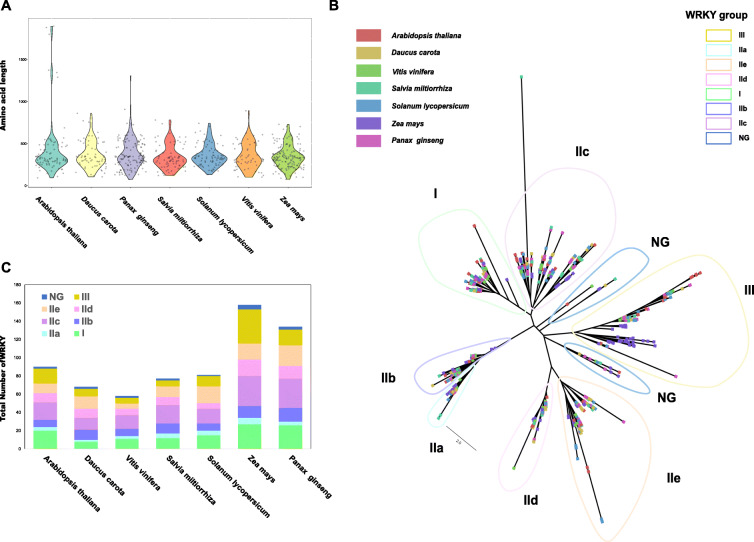
Phylogenetic analysis of the WRKY family in different plant species. **A** Length distribution of WRKY proteins in different plant species. **B** Unrooted NJ tree of WRKY proteins from seven plant species. **C **Distribution of WRKYs in different groups among seven species

To better understand the relationship of WRKYs in different species, WRKY genes from seven species were compared. Although the total number of WRKY genes between *A. thaliana*, *Daucus carota*, *Vitis vinifera*, *Salvia miltiorrhiza*, *Solanum lycopesicum*, *Zea mays* and *P. ginseng* was different, all seven species were remarkably similar regarding gene length distribution (Fig. 2 A). An unrooted ML phylogenetic tree was generated using the WRKYs from seven plant species to explore the evolutionary landscape of the WRKY genes across species (Fig. 2B). All of the WRKYs from the seven species were clustered into nine groups, the same seven classes (I, IIa, IIb, IIc, IId, IIe, III) as mentioned above and two NG groups. Although WRKY proteins could be stratified into the previously constructed groups clearly, the distribution of WRKY members was uneven in different groups (Fig. 2 C). This result is in accordance with a previous study in grapes and sunflowers [35, 36].

### Conserved motifs and gene structure analyses of ginseng WRKYs

**Fig. 3:**
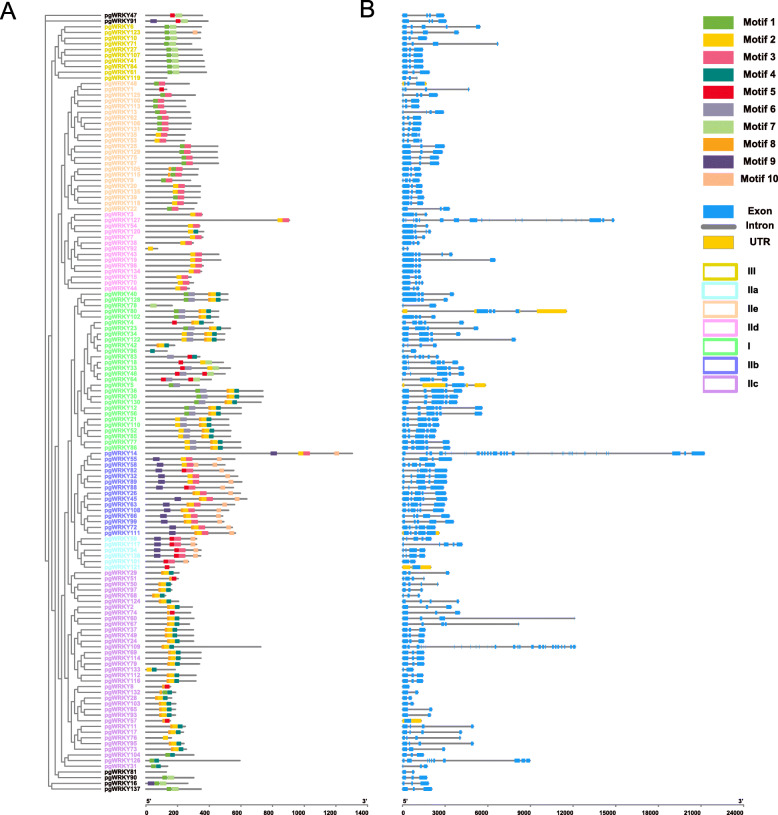
Conserved motif and gene structure analysis of PgWRKYs. **A** Motif distribution of PgWRKYs. **B** Gene structure of 137 PgWRKYs. The Motif composition of PgWRKY was analyzed by the MEME tool. The detailed information of the ten motifs is in Fig. S1

Ten individual motifs were predicted by the local MEME tool, revealing the distinct regions of PgWRKYs (Fig. 3 A). We annotated motif one and motif two as the classic WRKY DNA-binding domain (Fig. S1). The lengths of the ten motifs of PgWRKY ranged from 15 to 41 AA residues. Two PgWRKYs (PgWRKY81 and 119) did not have any motif, and the other 135 WRKYs varied from one to five motifs in each protein. In each subclass, the proteins harbor a similar number and type of motif, which suggests the functional similarities of these PgWRKYs.

To further examine the gene structure of PgWRKY, we constructed exon/intron structure diagrams based on the phylogenetic tree (Fig. 3B). All of the PgWRKY genes have at least one intron except for three genes (PgWRKY 57, 121 and 8). Among the 137 PgWRKYs, the majority had two introns and three exons, and a total of 52 PgWRKYs (37.9 %) had this type of structure. The structure type was three introns and four exons, and a total of 32 PgWRKYs (23.3 %) had this structure. The third structure type was four introns and five exons, and approximately 26 PgWRKYs (18.9 %) had this structure. Only three PgWRKYs, PgWRKY121, PgWRKY93 and PgWRKY8, did not have any introns (Fig. 3B). The exon numbers of PgWRKY genes within the same group were relatively similar.

### Cis-regulatory element analysis of the PgWRKY promoter

**Fig. 4:**
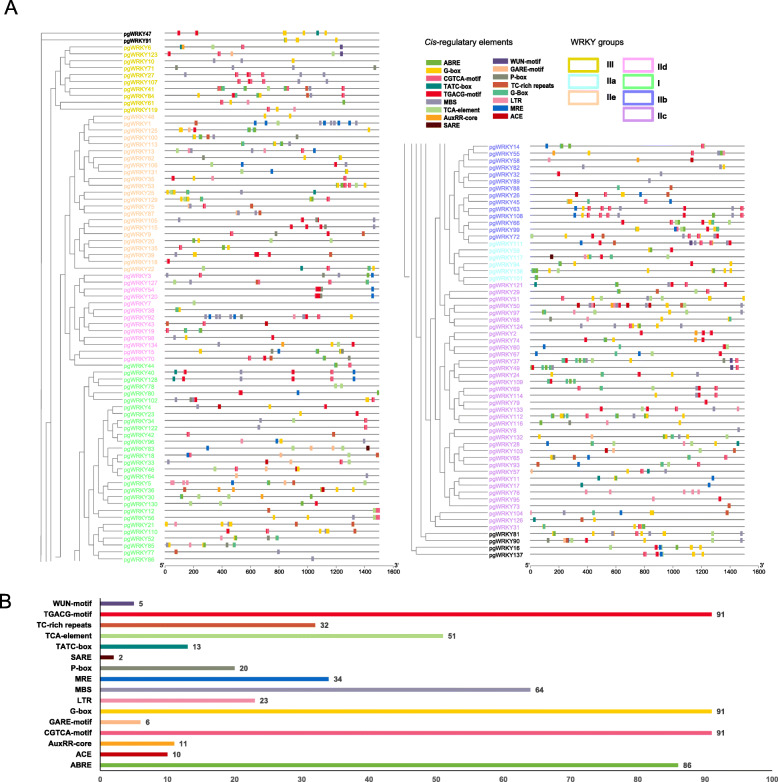
The cis-regulatory element prediction of PgWRKYs. **A **The distribution of cis-regulatory elements in PgWRKY promoters. **B** The distribution of different cis-regulatory elements in PgWRKY family

Cis-regulatory elements are the binding sites on the target gene for transcriptional modulation by transcription factors, and they are usually restricted to the 5’ upstream (promoter) sequence of the target gene. Herein, the 1.5 k upstream regions from the translation start sites of each PgWRKY were submitted to PlantCARE to survey stress-responsive cis-regulatory elements. A total of 15 stress response elements, consisting of TC-rich repeats (the cis-regulatory element for defense along with stress response), TATC-box (cis-regulatory element that participates in gibberellin-response), ACE (cis-regulatory element that engages in light response), LTR (cis-regulatory element that plays a role in low-temperature response), TCA-element (cis-regulatory element with a role in salicylic acid response), SARE (cis-regulatory element with a role in salicylic acid response), ABRE (cis-regulatory element associated with the abscisic acid response), AuxRR-core (cis-regulatory element with a role in auxin response), G-box (cis-regulatory element with a role in light response), CGTCA-motif (cis-regulatory element with a role in the MeJA-response), TGACG-motif (cis-regulatory element associated with MeJA-response), P-box (gibberellin-responsive element), GARE-motif (gibberellin-responsive element), WUN-motif (wound-responsive element), MBS (MYB binding site associated with drought-inducibility), and MRE (MYB binding site associated with light response), were identified (Fig. 4 A). All PgWRKYs had at least one stress response-linked cis-regulatory element. The cis-regulatory elements for hormone modulation consisting of CGTCA motifs, ABREs, AuxRR cores, P-boxes, TCA elements and TGA elements were also uncovered in numerous PgWRKY promoter regions. Overall, 87 PgWRKYs (80.8 %) had more than one ABRE motif, which indicated the prospective abscisic acid response under stress conditions. Approximately 91 PgWRKYs (70.2 %) had one or more CGTCA motifs that demonstrated the MeJA response potential, and the TCA element, TGACG motif, P-box, and AuxRR core were found in 51, 91, 20 and 11 PgWRKYs, respectively (Fig. 4B). 91G-box, 23 L, 64 MBS, and 32 TC-rich repeats were also found in PgWRKY promoter regions, which illustrated that these genes might play a role in cold, drought inducibility and defense responses.

### Expression of PgWRKY genes in different tissues

**Fig. 5:**
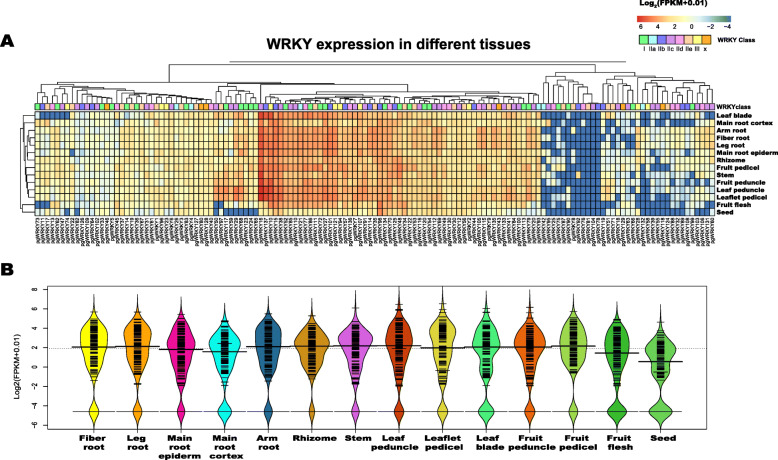
The expression of PgWRKY genes in ginseng tissues. **A** The heatmap of all PgWRKY genes expression in different tissues. **B** The beanplot of all PgWRKY genes expression in different tissues

The expression trends of PgWRKY genes in different tissues, such as stem, fiber root, fruit peduncle, main root epiderm, fruit pedicel, rhizome, leaf peduncle, arm root, leaflet pedicel, leg root, leaf blade, fruit flesh, main root cortex, and seed, were calculated by using FPKM values based on public RNA-seq data [37]. An expression heatmap of the WRKY genes was generated (Fig. 5 A). The gene expression results showed that only four PgWRKY genes (PgWRKY104, 78, 76 and 81; FPKM = 0) were not expressed in any tissue. The other 133 WRKY genes (97 %) were expressed in at least one tissue (FPKM ≥ 1), and approximately 60 (43.7 %) genes were expressed in all tissues (FPKM ≥ 1). The expression type of PgWRKYs could be divided into three groups: low-level expression, tissue-distinct and constitutive [36, 38]. Approximately 34 PgWRKY genes showed a low-level expression pattern in all tissues, and less than half of the genes showed tissue-distinct expression (Fig. 5 A). For instance, PgWRKY16 was expressed at a high level in each part of the root, rhizome, stem, leaf, and fruit but at a relatively low level in seed. The most highly expressed gene in seed and fruit flesh was PgWRKY98. Specifically, the WRKY genes exhibited relatively low-level expression in seeds (FPKM median is 1.72372, FPKM > 5, 46; FPKM > 10, 20). Over 58 % of PgWRKY genes showed marked expression (FPKM > 5), particularly in the fiber root, leg root, rhizome and fruit pedicel, and the highly expressed genes (FPKM > 10) were also distributed in these tissues (Table S2). The different expression trends of PgWRKYs indicated that these genes participate in diverse biological processes in various tissues.

### Expression of PgWRKY genes in response to abiotic stress

**Fig. 6:**
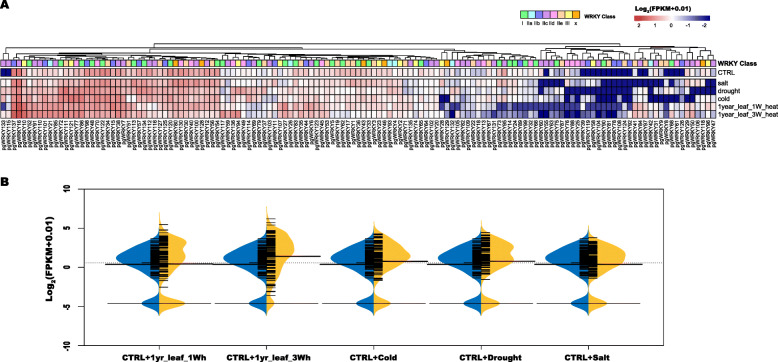
The expression of PgWRKY genes in response to different abiotic treatments. **A** The expression of PgWRKY genes for different abiotic treatments. **B** Beanplot of PgWRKY genes before and after abiotic stresses

The published data of different abiotic treatments on ginseng can provide more information for further study of the PgWRKY genes in response to abiotic stress. Cold, salt, drought and heat treatments were applied in a previous study [30]. As illustrated in Fig. 6, many PgWRKY genes showed a similar changing expression trend after abiotic stress treatment. The results showed that 14 PgWRKYs (PgWRKY22, -25, -58, -63, -66, -81, -88, -89, -90, -104, -108, -116, -124, -129 FPKM<1) were not expressed in any groups, and two of these genes (PgWRKY104 and 81) were also not expressed in any tissue, as described in the former sections. The PgWRKYs showed different response patterns for different treatments. In the heat treatment, 25 PgWRKYs (fold change > 2) were found to have significant changes between the control and one-week heat treatment, and four of these PgWRKYs (PgWRKY91, -51, -6 and -101) had more than four-fold changes. Approximately 45 PgWRKYs (fold change > 2) were found to have significant changes between the control and three-week heat treatment, 26 PgWRKYs had more than four-fold changes, and the most changed gene was PgWRKY136, which had 136-fold changes. In total, 26 PgWRKYs were found to change in both one week and three weeks of treatment, and detailed information on the heat treatment results is shown in Table S3. The expression trend analysis indicated that many PgWRKYs might be involved in the response to heat treatment at three weeks (Fig. 6B, Table S4).

The cold treatment caused 16 genes to change (fold change > 2), and most of them were upregulated. Approximately 11 of them changed over four-fold. PgWRKY133 was found to be expressed only in cold-treated samples. A total of 22 PgWRKYs (fold change > 2) were found to respond to drought treatment, and approximately 12 of them had over four-fold changes. PgWRKY72 exhibited an approximately 30-fold change after drought treatment, and two WRKYs (PgWRKY115 and PgWRKY133) were found to be expressed only in drought-treated samples. Interestingly, no PgWRKY was found to be downregulated after drought treatment, and this result was in accordance with a previous report showing that upregulation of numerous TFs under water stress may result in enhanced expression of target genes [39, 40]. The salt treatment only caused Four PgWRKYs (fold change > 2) to change significantly: PgWRKY80, PgWRKY100, PgWRKY111 and PgWRKY113. Only PgWRKY113 changed approximately five-fold, and the other PgWRKYs changed slightly. These results illustrated that PgWRKY might not respond to salt in ginseng. The expression trends of genes changed significantly under drought and cold treatment but slightly in response to salt treatment (Fig. 6B, Table S5).

### Coexpression analysis of candidate ginsenoside biosynthesis PgWRKY genes

**Fig. 7:**
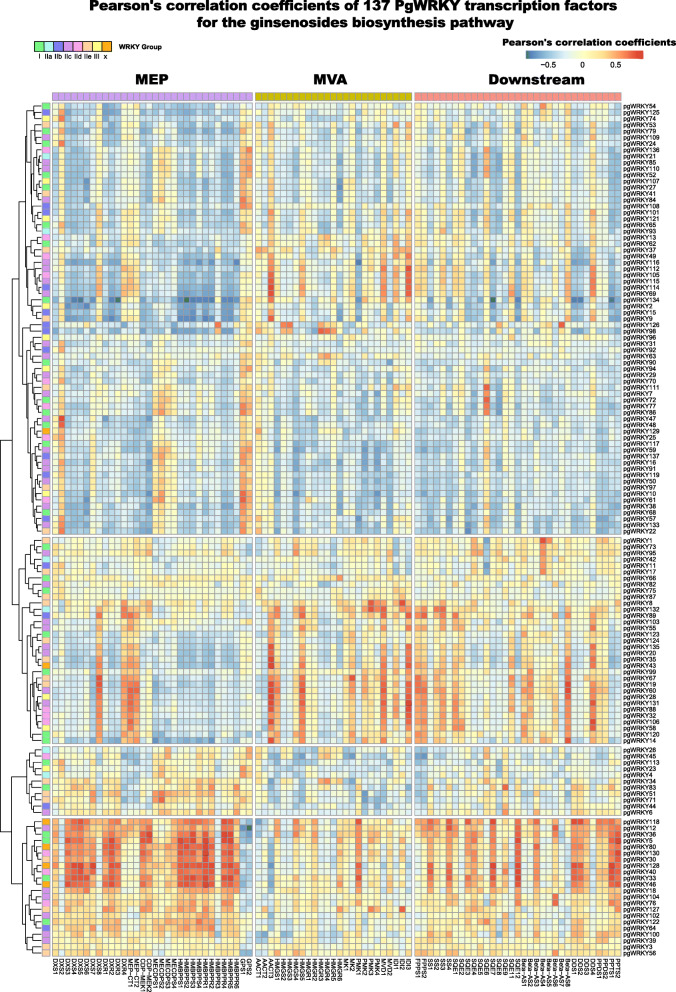
The Pearson’s correlation coefficients of PgWRKYs with ginsenoside biosynthesis pathway. Protopanaxatriol synthase (PPTS); Protopanaxadiol synthase (PPDS); Dammarenediol II synthase (DDS); βamyrin synthase (β-AS); Squalene epoxidase (SQE); Squalene synthase (SS); Farnesyl diphosphate synthase (FPPS); Isopentenyl-diphosphate delta-isomerase (IDI); Mevalonate diphosphate decarboxylase (MVD); Phosphomevalonate kinase (PMK); Mevalonate kinase (MK); 3-hydroxy-3-methylglutaryl-CoA synthase (HMGS); 3-hydroxy-3- methylglutaryl-CoA reductase (HMGR); Acetyl-CoA C-acetyltransferase (AACT); Geranyl diphosphate synthase (GPS); 4-hydroxy-3-methylbut-2-en-1-yl diphosphate reductase (HMBPPR); (E)-4-hydroxy-3-methylbut-2-enyl-diphosphate synthase (HMBPPS); 2-C-methyl-D-erythritol 2,4-cyclodiphosphate synthase (MECDPS); 4-diphosphocytidyl-2-C-methyl-D-erythritol kinase (CDP-MEK); 2-C-methyl-D-erythritol 4-phosphate cytidylyltransferase (MEP-CT); DXP reductoisomerase (DXR); 1-deoxy-D-xylulose-5-phosphate synthase (DXS)

WRKY participates in numerous physiological activities of plants. To elucidate the relationship of PgWRKYs to ginsenoside biosynthesis, we generated a coexpression network of PgWRKYs and candidate ginsenoside biosynthesis genes consisting of the upstream MEP, MVA pathway and downstream saponin skeleton formation pathway (Fig. 7). The coexpression analysis results showed that the correlation between PgWRKYs and ginsenoside biosynthesis-related genes could be divided into four clusters. PgWRKYs in Cluster I have a lower correlation with ginsenoside biosynthesis pathway genes. Only a few PgWRKYs (PgWRKY112, -105, -114, -115) in this cluster have a higher correlation with IDI3 and HMGS1 of the MVA pathway. In Cluster II, multiple PgWRKYs showed a strong correlation with MEP pathway upstream genes, such as DXS8, DXR4, MEP-CT1, and MEP-CT2. Other genes in the MEP pathway did not show a positive correlation with Cluster II PgWRKYs. The PgWRKYs in Cluster II also showed a strong correlation with some genes in the MVA and downstream pathways. For instance, strong correlations were found in some PgWRKYs with AACT2, HMGS5, MK1, PMK2, MVD1, IDI2, and IDI3 in the MVA pathway. The Cluster III group WRKY did not show any positive correlation with genes of the ginsenoside biosynthesis pathway. Many PgWRKYs in Cluster IV have strong positive correlations with genes in the MEP and downstream pathways. The genes named DXS, DXR, CDP-MEK, HMBPPR and HMBPPS in the MEP pathway have strong positive correlations with cluster IV PgWRKYs. SS, SQE, Beta-AS, DDS and PPTs in the downstream pathway also have a strong correlation with cluster IV PgWRKYs. These results indicate that PgWRKYs in Cluster IV might participate in the modulation of ginsenoside biosynthesis. The results of coexpression analysis indicated that the MVA pathway genes were correlated to Cluster II PgWRKYs, and the MEP pathway genes were correlated to Cluster IV PgWRKYs. A total of 11 genes named PgWRKY118, -12, -36, -5, -80, -130, -30, -128, -40, -33 and -46 were selected to have modulatory potential for ginsenoside biosynthesis. The r value of the correlation between these PgWRKY genes and ginsenoside biosynthesis pathway genes was greater than 0.7 (Table S6).

**Fig. 8:**
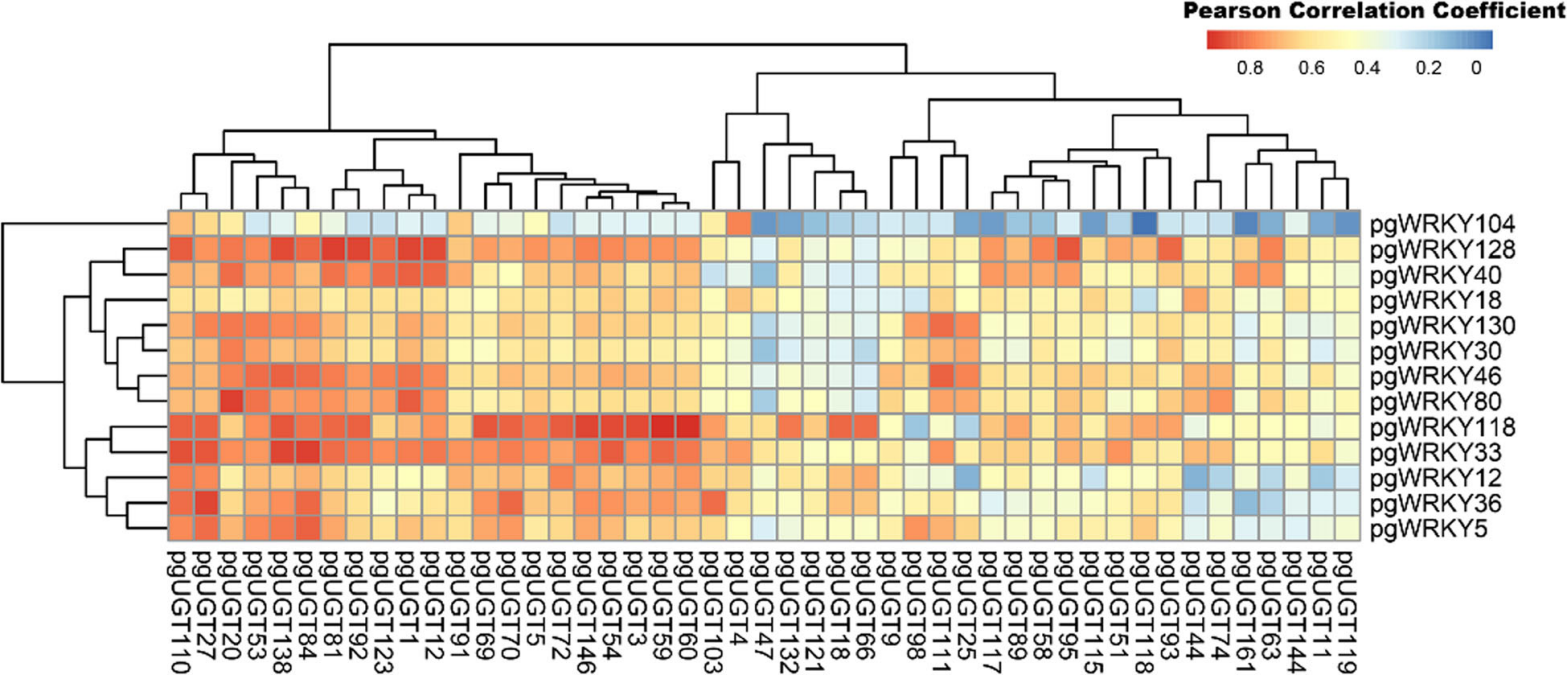
The Pearson’s correlation coefficients of selected PgWRKYs with ginseng UGTs

Glycosyltransferases usually play an indispensable role in the biosynthesis of triterpenoids [41]. In the ginsenoside biosynthesis pathway, glycosyltransferases usually catalyze the final steps to form different ginsenosides as tailed enzymes [42]. To further confirm the correlation between the PgWRKYs and the final stages of ginsenoside biosynthesis, we selected 155 ginseng glycosyltransferases (Table S7) from the ginseng genome and calculated the correlation between the selected 11 PgWRKYs and the 155 PgUGTs (Table S8). We found that 47 PgUGTs had a positive correlation (r > 0) with these 11 PgWRKYs (Fig. 8). Finally, a total of 24 UGTs were found to have a strong correlation with these 11 PgWRKYs (r > 0.8). To further annotate these 24 PgUGTs, we found that all of the reported ginsenoside biosynthesis-related UGTs were included in the 24 PgUGTs [43–46] (Table S9). This result indicates that these 11 PgWRKYs have strong potential in the regulation of UGTs in the ginsenoside biosynthesis pathway.

## Discussion

The WRKY family is a pivotal and large transcription factor family in higher plants. Studies of the WRKY family have been performed in many plants, consisting of model plants, important crops and some medicinal plants, such as Arabidopsis (*A. thaliana*) [47], sesame (*Sesamum indicum*) [48], pineapple (*Ananas comosus*) [49], sunflower (*Helianthus annuus*) [36], licorice (*Glycyrrhiza glabra*) [50], carrot (*Daucas carota*) [51] and chickpea (*Cicer arietinum*) [52]. Herein, a total of 137 PgWRKY genes were obtained from the ginseng genome by bioinformatics analysis.

The members of the WRKY family in different plants were uneven. As shown in Fig. 2 A, among the seven species, the WRKY members ranged from 59 to 161 (*Z. mays*, 161, genome size 2100 Mb; *P. ginseng*, 137, genome size 2900 Mb; *A. thaliana*, 90, genome size 121 Mb; *D. carota*, 69, genome size 421 Mb; *S. miltiorrhiza*, 77, genome size 547 Mb; *S. Lycopersicum*, 81, genome size 809 Mb and *V. vinifera*, 59, genome size 427 Mb). The genome sizes of these plants ranged from 119.75 Mb to 2.98 Gb, *Z. mays* had the most prominent WRKY family members, and a minor WRKY family was found in *V. vinifera*. Ginseng has the second largest WRKY family members, although the genome size of ginseng is larger than that of *Z. mays*. This result indicated that the number of WRKY genes was not associated with genome size. A previous study reported that WRKY family expansion has occurred in maize (*Z. mays*) and rice (*O. sativa*) [53, 54]. The ginseng genome experienced two whole-genome duplication (WGD) events at 2.2 Million Years and 28 Million Years Ago [30]. TFs are usually retained after the WGD event [55], suggesting that WGD might be a major reason for WRKY family expansion in ginseng.

PgWRKYs were grouped based on the conserved WRKY domain along with the zinc-finger motif. Three primary groups (I, II, III) and five subgroups (IIa, IIb, IIc, IId, IIe) were established by alignment. There were 27 PgWRKYs in Group I, 95 in group II, and 10 in group III. The largest group of PgWRKY was group II. The distribution of WRKY genes in groups among plant species was different. Ginseng (137) has a large group II(95) and a relatively small group III(10), and a similar WRKY distribution was found in grape [56] and eggplant [57]. In Arabidopsis (*A. thaliana*), maize (*Z. mays*) and rice (*O. sativa*), group III is relatively large compared to that in other plants [4, 53, 58]. Group III WRKY TFs are thought to play a vital role in plant evolution and adaptation. Due to the fewer group III genes compared to other large genome plants, the expansion of the PgWRKY family in ginseng might be caused by the expansion of other WRKY groups [59].

The WRKY domain is highly conserved in most plants with the heptapeptide motif WRKYGQK. The conserved heptapeptide motif WRKYGQK was found in most of the PgWRKYs. However, variants such as WRKYDQK, WRKYGKK and WKKYGKK were also found in some PgWRKYs. WRKYGKK appeared in six PgWRKYs, five of which (PgWRKY29, -51, -50, -97, and -124) belonged to group IIc, and only one (PgWRKY125) belonged to group IIe. Two PgWRKYs (PgWRKY15 and -70) have WKKYDQK, and they belong to IId. PgWRKY44 has WRKYDQK, PgWRKY68 has WKKYGKK, and they both belong to IIe. Variants were also found in *Asteranae*, *Nelumbo nucifera* and apple [60–62]. Previous studies reported that variation in the WRKY domain might alter the DNA binding ability. NtWRKY12 (*Nicotiana tabacum*), GmWRKY6 and GmWRKY21 (*G. max*) showed more WK box (TTTTCCAC) binding ability than the W box (TTGACC) for WRKYGKK motifs [63, 64].

Further analysis of PgWRKYs showed differential distribution patterns of cis-regulatory elements in different PgWRKY promoters, which suggests that these PgWRKYs regulate diverse biological processes of plant growth during development. Numerous abiotic stress-distinct cis-regulatory elements have been identified in the PgWRKY promoter region (Fig. 4). These different kinds of cis-regulatory elements in their promoter regions might be related to the long-term evolution and environmental adaptability of ginseng. In addition, the existence of diverse cis-regulatory elements that respond to plant hormones (ABA, MeJA, and SA) indicates that they are involved in regulating various hormone signaling pathways related to ginseng adaptation to abiotic stress along with biotic stress.

A variety of PgWRKY promoters have hormone and MeJA response elements. PgWRKY54 and PgWRKY69 contain MeJA-responsive cis-regulatory elements, and a previous study of ginseng WRKY showed that these two genes were significantly downregulated after MeJA treatment [33]. PgWRKY100, PgWRKY69, PgWRKY131 and PgWRKY35 contain ABRE elements that are associated with the abscisic acid response. A previous study showed that all four PgWRKYs responded to abscisic acid treatment [33]. A total of 64 PgWRKYs have one MBS element, the MYB binding site associated with drought inducibility. Compared with the drought treatment results, we found that 15 of the 64 PgWRKYs were significantly changed. The results of cis-regulatory element analysis could help to predict the potential function of PgWRKYs for further study.

The expression levels of PgWRKYs in 14 different tissues were calculated based on FPKM. As shown in Fig. 5 A, PgWRKYs showed diverse expression patterns in the 14 tissues. This result indicated that PgWRKYs might perform diversified functions during the ginseng lifespan. Among all 137 PgWRKY genes, only three PgWRKY genes were not expressed in any tissues, 134 PgWRKYs were expressed in at least one tissue (FPKM > 1), and their expression levels were altered significantly among 14 tissues. As shown in Fig. 5B, the rhizome, stem, leaf peduncle and fruit pedicel had higher median FPKM values than other tissues, which indicated that PgWRKY might participate in more physiological processes in these tissues. PgWRKY16 is expressed predominantly in almost every tissue except fruit flesh and seed. PgWRKY10 was highly expressed in aerial parts, including fruit flesh. PgWRKY137 was highly expressed in aerial parts, such as PgWRKY10, but expressed at low levels in fruit flesh. PgWRKY98 is only highly expressed in fruit flesh and seed. A set of PgWRKYs, including PgWRKY15, -137, -101, -10, -121 and -38, was highly expressed in root tissues. These tissue-specific expression patterns suggest that these PgWRKYs might be involved in tissue-specific development and signaling processes. Interestingly, some PgWRKYs showed different expression in the main root epiderm and cortex, and most of the expressed PgWRKYs were highly expressed in the epiderm. Since the ginseng epiderm is in direct contact with the external environment, the highly expressed genes in the epiderm are more likely to participate in the environmental stress response. PgWRKY111 was highly expressed in the epiderm and responded to all abiotic stresses in this study. The ortholog of PgWRKY111 is AtWRKY33, which was found to be involved in the response to cold and salt stress [65]. Both PgWRKY11 and -17 were highly expressed in the cortex and extremely low in the epiderm. The ortholog of these two genes in Arabidopsis is AtWRKY12, which can regulate secondary cell wall formation in Arabidopsis [66]. This result indicated that both PgWRKY11 and -17 may also be involved in secondary cell wall formation in the cortex. Further study of the tissue-specific expressed PgWRKYs could provide crucial information for understanding the function of the ginseng WRKY gene family.

During a long period of evolution, plants have to face complex environmental factors, such as drought, salt, heat or cold, and other uncertain ecological changes [67]. Previous studies have demonstrated the important roles of WRKY transcription factors in the response to abiotic stress in plants [47, 52, 67, 68] In this study, a total of 54 PgWRKY genes were induced under different abiotic stresses. For instance, 15 PgWRKYs responded to cold treatment, 22 PgWRKYs responded to drought treatment, three PgWRKYs changed under salt treatment, 25 PgWRKYs changed under one-week high temperature, and 45 PgWRKYs changed under three-week high temperature. Only PgWRKY111 responded to all treatments, and another five PgWRKYs, namely, PgWRKY121, -70, -85, -52, -72 and -86, responded to cold, drought and heat treatments. These WRKYs might be the central response TFs for abiotic stress. Overexpression of *Os*WRKY11 and *Os*WRKY45 could lead to enhanced heat, drought and salt and tolerance [24, 69]. In soybean (*G. max*), overexpression of GmWRKY21 and GmWRKY54 shows enhanced cold, salt and drought tolerance [64]. These PgWRKYs have the potential to be the regulatory point for enhancing the tolerance of abiotic stress in ginseng.

WRKYs are involved in an extensive range of plant physiological development processes and affect the biosynthesis of secondary metabolites. A previous study reported that overexpression of *Cb*WRKY24 could promote saponin accumulation in *Conyza blinii* [70], and AvWRKYs could activate the promoter of AvNeoD involved in the saponin biosynthesis pathway in *Amomum villosum* [71]. In our study, we found that 13 PgWRKYs were positively correlated with ginsenoside biosynthesis pathway genes. These PgWRKY genes are distributed in phylogenetic Groups I and II. There were 11 PgWRKYs in phylogenetic Group I and only two PgWRKYs in phylogenetic group II. One is in subgroup IIc (PgWRKY104), and the other is in subgroup IIe (PgWRKY118). Further analysis revealed that the selected PgWRKYs were more highly related to PPT-type ginsenoside pathway genes. For example, PgWRKY5, -130, -128, -40 and -33 have a high correlation (r > 0.8) with PPTS1 and PPTS2, which are the candidate key enzymes that catalyze the branch point of PPD-type and PPT-type ginsenosides. These coexpression relationships reflect the modulation of ginsenoside biosynthesis by PgWRKYs, and further overexpression or knockout analysis of these PgWRKY genes may help to elucidate their function.

## Conclusions

In this study, genome-wide identification of WRKY TFs in ginseng and their expression in different tissues and responses to different abiotic stresses were performed. Coexpression analysis revealed the potential regulatory relationship of PgWRKYs to ginsenoside biosynthesis pathway genes. Our findings suggest a foundation for further functional study of the regulatory mechanism of PgWRKYs in plant stress responses and provide more valuable information for Ginseng breeding and metabolic engineering.

## Methods

### Sequence retrieval and identification

The candidate WRKY genes were firstly obtained from the Ginseng Genome Data resource (http://ginsengdb.snu.ac.kr/) [72]. The WRKY Hidden Markov Model (HMM) profile (PF03106) was abstracted from Pfam data resource (http://pfam.xfam.org). The HMMER 3.2.1 software was employed to check the WRKY genes retrieved from the ginseng genome, and the E-value threshold was 10−2. All candidate PgWRKYs were further validated by using the SMART data resource (http://smart.embl.de/) along with the NCBI-Conserved Domain Database (CCD) to ensure that they contained the WRKY domains. The WRKY data sets of *A. thaliana*, *S. lycopersicum*, *D. carota*, *S. miltiorrhiza*, *V. vinifera* and *Z. mays* were gained from the PlantTFDB (Plant Transcription Factor Database) (http://planttfdb.cbi.pku.edu.cn).

ClustalW with the default parameters was used for Multiple WRKY sequences alignments between ginseng and other species. The IQ-TREE was utilized by using the Maximum Likelihood approach based on the LG+I+G model [73] to establish the ginseng WRKY phylogenetic tree, and the nodes were tested by bootstrap analysis with 1000 replicates. For WRKYs phylogenetic tree between different species, MEGAX was used to make the tree by using the Neighbour-joining approach and 1000 bootstrap replications. The further annotation of the phylogenetic tree result was processed by iTOL (http://itol.embl.de).

### Conserved motifs and gene structure analysis

The gene structure was abstracted from the ginseng genome annotation file (http://ginsengdb.snu.ac.kr/). TBtools 1.053 was employed to demonstrate the gene structure [74]. The conserved motifs of PgWRKYs were identified by using MEME local software (version 4.12.0) in Linux with the following parameters: maximum of 10 misfits and an optimum motif width of 6 - 100 amino acid residues. In addition, theoretical isoelectric point (pI) along with the molecular weight (MW) of PgWRKY proteins were predicted by the online Sequence Manipulation Suite (http://www.detaibio.com/sms2/reference.html) [75].

### PgWRKY gene expression analysis

For analyzing gene expression among the different tissues and the response for different abiotic treatment. 14 RNA-Seq datasets of different tissues from NCBI (accession number PRJNA302556) and 15 RNA-Seq datasets for abiotic treatment ( No.24-38 in ginseng transcriptome data resource, http://ginsengdb.snu.ac.kr/ transcriptome.php ) from Ginseng Genome Data Resource ( http://ginsengdb.snu.ac.kr/ ) were retrieved. The clean reads were aligned to the ginseng genome with Hisat2 software. Cufflinks along with Cuffmerge were used to assemble and calculate the expression value for each transcript. The fragments per kilobase of exon per million mapped reads (FRKM) method were used to identify differentially expressed genes (DEGs) among the different samples [76]. The R package “Hmisc” was employed to compute the Pearson’s correlation between the ginsenoside pathway genes and PgWRKYs in the 14 RNA-Seq tissues expression datasets. The abiotic treatment mothed was like this, the one-year-old ginseng was inoculated with 100 mM NaCl solution for 24 h for salt stress; maintained at 4 °C for 24 h for cold treatment, removed from the soil and air-dried on 3MM paper for 24 h for drought treatment and treated with 30 (±1)°C for one week and three weeks for heat treatment [30].

## Supplementary information

Additional file 1 **Figure S1**: The detail information of Motifs.

Additional file 2 **Table S1**: WRKY information, **Table S2**: The expression of WRKY in different tissues, **Table S3**: The expression of WRKY under different treatments, **Table S4**: The different expression genes under heat treatment, **Table S5**: The different expression genes under cold, salt and drought treatment, **Table S6**: the correlation matrix of WRKY to ginsenosides pathway genes, **Table S7**: the expression of UGTs in P. ginseng, **Table S8**: The correlation matrix of WRKY to select PgUGTs, **Table S9**: The annotation of select PgUGTs.

## Data Availability

The raw RNA-Seq data of *P. ginseng* stem, fiber root, fruit peduncle, main root epiderm, fruit pedicel, rhizome, leaf peduncle, arm root, leaflet pedicel, leg root, leaf blade, fruit flesh, main root cortex, and seed were downloaded from the NCBI Sequence Read Archive ( https://www.ncbi.nlm.nih.gov/bioproject/PRJNA302556 ) (NCBI Sequence Read Archive SRR2952867, SRR2952868, SRR2952869, SRR2952870, SRR2952871, SRR2952872, SRR2952873, SRR2952874, SRR2952875, SRR2952876, SRR2952877, SRR2952878, SRR2952879, SRR2952880) [37]. The raw RNA-Seq datasets of drought, salt and cold treatment were downloaded from Ginseng Genome Database ( http://ginsengdb.snu.ac.kr/download.php?filename=DSC.tar.gz ) and the heat stress were downloaded from Ginseng Genome Database ( http://ginsengdb.snu.ac.kr/download.php?filename=Heat.tar.gz ) [72]. The sequences of candidate *P. ginseng* WRKY genes used in this study were download from the Ginseng Genome Database ( http://ginsengdb.snu.ac.kr/tf_list_2.php?id=WRKY ). The *P. ginseng* UGT gene sequences used in this study were download from the Ginseng Genome Database ( http://ginsengdb.snu.ac.kr/gene_annotation.php ). All other data generated or analyzed in this study are included in this article and its additional files.

## Abbreviations

TF: Transcription factor
CDS: coding sequences
FPKM: Fragments per kilobase of transcript per million mapped fragments
MW: Molecular weight
PI: Isoelectric point
AA: amino acid
ML: maximum likelihood
MEP: 2-C-methyl-d-erythritol 4-phosphate
MVA: mevalonate
PPTS: Protopanaxatriol synthase
PPDS: Protopanaxadiol synthase
DDS: Dammarenediol II synthase
β-AS: βamyrin synthase
SQE: Squalene epoxidase
SS: Squalene synthase
FPPS: Farnesyl diphosphate synthase
IDI: Isopentenyl-diphosphate delta-isomerase
MVD: Mevalonate diphosphate decarboxylase
PMK: Phosphomevalonate kinase
MK: mevalonate kinase
HMGS: 3-hydroxy-3-methylglutaryl-CoA synthase
HMGR: 3-hydroxy-3- methylglutaryl-CoA reductase
AACT: acetyl-CoA C-acetyltransferase
GPS: geranyl diphosphate synthase
HMBPPR: 4-hydroxy-3-methylbut-2-en-1-yl diphosphate reductase
HMBPPS: 4-hydroxy-3-methylbut-2-enyl-diphosphate synthase
MECDPS: 2-C-methyl-D-erythritol 2,4-cyclodiphosphate synthase
CDP-MEK: 4-diphosphocytidyl-2-C-methyl-D-erythritol kinase
MEP-CT: 2-C-methyl-D-erythritol 4-phosphate cytidylyltransferase
DXR: DXP reductoisomerase
DXS: 1-deoxy-D-xylulose-5-phosphate synthase
WGD: whole-genome duplication
