# Supplementary figures and images for: Genome-wide characterization and analysis of WRKY transcription factors in Panax ginseng
Source: BMC Genomics. 2021 Nov 18;22:834. doi: 10.1186/s12864-021-08145-5 (PMC8600734; doi:10.1186/s12864-021-08145-5)

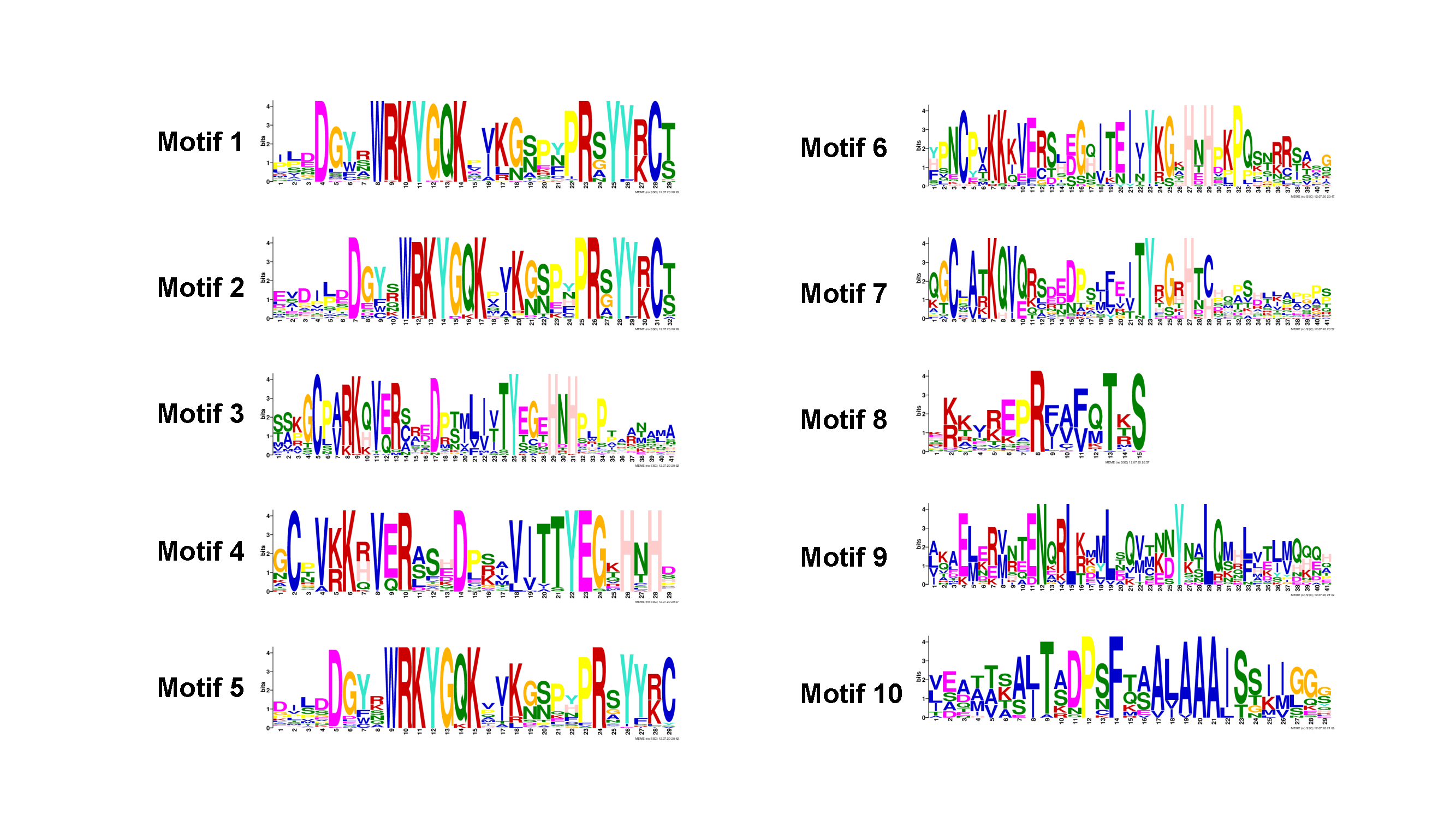

Supplement: Supplementary file 1 — Figure S1: The detail information of Motifs. [file 12864_2021_8145_MOESM1_ESM.png]
